# Procidentia or Prolapsus Uteri*Read before the Fifth and Sixth District Medical Society at Corpus Christi, Texas, September 7-8, 1914.

**Published:** 1914-11

**Authors:** P. D. Spohn

**Affiliations:** Corpus Christi, Texas


### TEXAS MEDICAL JOURNAL

**Dr. F. E. Daniel, Founder. Established July, 1885**

MRS. F. E. DANIEL, - Publisher and Managing Editor

Published Monthly.—Subscription, $1.00 a Year.

VOL. XXX AUSTIN, NOVEMBER, 1914 No. 5

The publisher is not responsible for views of contributors

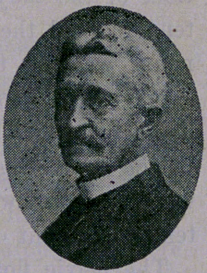

### Original Articles.

For Texas Medical Journal.

### Procidentia or Prolapsus Uteri.*

BY P. D. SPOHN, M. D., CORPUS CHRISTI, TEXAS.

My paper is entitled "Procidentia or Prolapsus Uteri," which is defined as a falling down of the womb which in descending invests the vagina, owing to the relaxation of its connections. When it protrudes through the external parts, it is in the extreme degree of displacement and is termed complete prolapse. I would like first to run over the anatomy of the uterus, its adnexa, and its muscles and ligaments.

The normal uterus is a movable organ swung by its supports in the pelvic cavity between the abdominal cavity proper and the pelvic floor. It is held in position: (1) Above, by the retentive power of the abdominal cavity; (2) below, by the pelvic floor; (3) by the adjacent pelvic organs; (4) its relation which its axis bears to the axis of the vagina.

In the erect position with an empty bladder and rectum the external os is in the plane of the ischial spines, or somewhat below it, and a little posterior to the center of the pelvis. The cervix projects into the vagina pointing towards the last sacral vertebra, held in position by the utero-pelvic and utero-sacral ligaments and the fascia between the bladder and uterus. The axis of the uterus lies in different planes, according to the fullness of the bladder and rectum, lying almost horizontally when the bladder is empty. The attachments also have a yielding character and allow move-

*Read before the Fifth and Sixth District Medical Society at Corpus Christi, Texas, September 7-8, 1914.

ment in many directions during respiration and changing positions of the body. The round ligaments are the guy ropes which tend to hold the uterus forward. In the base of the broad ligament, we find the utero-pelvic ligament running out from the supra-vaginal portion of the cervix to the pelvic fascia, and these ligaments control the lateral movements of the uterus.

The broad ligaments themselves help to hold the cervix upwards and backwards, as also do the utero-sacral ligaments, which tend to prevent downward displacement.

The pelvic floor in supporting all the pelvic organs indirectly supports the uterus, and with the utero-sacral and utero-pelvic ligaments forms the main barrier to downward displacement. Nature has given to every woman these supports, and when from any cause or accident they lose their efficiency something nearly always happens, and that something is a prolapsus uteri.

In discussing the causes of prolapse, I do not intend to discuss cases that are secondary to other pelvic lesions, such as pelvic tumors, for these conditions which produce the symptoms and the prolapse are only a result of another abnormality. However, if these conditions have existed long enough, the pelvic organs may lose their retentive power and the uterine supports become weakened and stretched. In these cases, even if the cause of the prolapse be removed, the uterus will probably remain in a condition of prolapse, due to its weakened and insufficient supports.

Laceration of the perineum is probably the greatest cause of prolapse. Defecation is more difficult, as the support and action of the levator ani is lessened, and thus extra force is exerted to empty the rectum. Thus with the perineum and levator ani torn the feces are driven against the posterior vaginal wall, which in time produces a rectocele which also pulls on the cervix and uterus. We can easily see how in time other structures, such as the bladder, become prolapsed from the abnormal pressure from behind, producing a prolapse of the anterior vaginal wall. Thus we see when one support is gone or inefficient the other supports are put on the stretch.

In abnormalities of the pelvis, especially when the organs are not in close apposition, the supports are more liable to allow a greater degree of movement and allow the uterus to sag. In chronic constipation, with an overloaded bowel, the cervix is pushed downward and backward until the contents are expelled. This keeps up a constant strain on the utero-sacral-ligaments, and this constant tension, in time, will weaken the ligaments, especially in women.

Subinvolution following labor or miscarriage may cause prolapse, as in addition to the increased size of the uterus the vagina and ligaments are also in a state of subinvolution.

Different postures and occupations, especially in women who are in a bad state of health, are liable to predispose to prolapse. Any condition that may cause a retroversion or anteversion may indirectly result in a prolapse. In pulmonary tuberculosis, where there is persistent coughing, the uterine ligaments are sometimes unable to stand the extra intra-abdominal pressure put upon them as they are already in a weakened condition and have lost some of their resisting power.

Deformities of the vertebral column from Pott's disease may change the angle of the intra-abdominal pressure on the floor of the pelvis, and may also favor prolapse.

I suppose there is no commoner phrase used by the quack doctor to his female patients than "crooked womb" or female trouble. Whether his examination is superficial or done in a fairly thorough manner, his patient will go out and tell the neighbors that she has a crooked womb and Dr. So-and-So has to perform an operation. Of course, the operation will cure her and relieve her of her symptoms, especially if the doctor is paid in advance. The patient may need an operation all right, but in a good many cases, whether the trouble be anteversion, retroversion, prolapse or anything else, the operation is almost sure to be a curettage, and nothing more. This is done by others than quacks who really may believe they are doing the right thing, when they have not made a careful enough examination and have not diagnosed the cause of the trouble. A thorough examination should be made in each case, and the physician should be sure he is not dealing with an inverted uterus, an hypertrophied cervix or a cervical polypus.

Backache is, I suppose, the commonest symptom, and is generally referred to lumbar region or over the upper part of the sacrum. It is more or less relieved when the patient lies down and increased in the erect posture, especially when doing heavy work. A bearing down or a dragging weight in the pelvis with pains referred down the thighs. The rectal symptoms I put down are constipation, sensation of fullness in the bowel, difficult defecation and perhaps hemorrhoids.

The bladder in severe cases is likely to show symptoms of irritation and difficult urination. There is always a leucorrhea, with a discharge of a whitish or whitish-yellow color, due to the congestive endometritis.

Menstruation is generally more profuse. While many women suffering from prolapse do not become pregnant, many others go on to the full term, with the condition always returning after labor; other cases miscarry frequently.

I know of one case with a very marked prolapse who has had six children and five miscarriages. This patient has refused operation at different times, and I would like to give a little illustration of her case as a point of interest, especially to show the condition of the uterine wall. I was called to see her some six weeks after she had a miscarriage, and found that there was quite a flow of blood from the uterus each day, which I diagnosed was due to retained membranes, etc., and which stopped when I did a curettage. This patient had a profuse leucorrhea tears in both the cervix and perineum and a very marked prolapse. I might state to defend myself in one sense I did not attend her at any of her miscarriages or confinements. The point I want to bring out is the soft condition of the uterine wall, which I found out to my great sorrow. I think I never did a curettement more carefully, and I was cautious or thought I was cautious about using any force on the instrument. Well, to come to the point, all at once I felt that curette slip up about four inches, and I recognized at once what I had done. I may say that I didn't feel very well, but I did not say anything to the anesthetist until afterward. Anyway, I felt mighty anxious for three or four days, as I certainly punctured that uterus. Fortunately, no harm came from it, as her temperature remained normal, and she made an uninterrupted recovery. The point I want to illustrate is that the uterine wall was extremely soft, and I think it was due to the extreme prolapse, the whole organ being in a state of atony. The condition was probably somewhat increased by the retention of the membranes.

The whole general condition of a woman suffering from prolapse is weakened, and she has symptoms of nervousness and disorders of digestion.

In bringing up the treatment of the prolapse of the uterus, I am just going to give a brief description of some of the operations, and hope to start a discussion on the merits of the procedure. In passing, I would like to mention the use of pessaries.

Occasionally, a round, hard rubber or soft rubber ring pessary will be retained in the vagina and will afford a certain amount of support to the prolapsed bladder and uterus and give considerable relief. The various cup and stem pessaries attached to abdominal belts, though, that may hold up the uterus usually produce more discomfort than the prolapse itself.

Prolapsus uteri is not an uncommon trouble in elderly women, due to the fact that many women refuse to submit to local or operative treatment, and to the neglect of the profession to recommend operative treatment when indicated. Many women have suffered from this for years, and we may be called upon to try to improve the condition rather than to cure it.

Under non-operative treatment, I will just mention the use of tampons, hot douches and putting the patient in the knee-chest position for a certain time each day, all of which do a certain amount of good when there is no other serious pathological condition. It is also, I believe, good practice to give a preliminary treatment of douches and tampons for awhile previous to operation. The general condition of the patient should be rendered as healthy as possible in each case before operative treatment is instituted.

I want to touch on a few points of the operations. Ventral suspension is only suitable for patients who have passed the menopause or who have been rendered sterile at the time of operation. In younger women, when pregnancy occurs, there is likelihood of abortion, and if pregnancy reaches the full term dangerous obstacles present themselves to the delivery of the child. Then the resulting attachment of uterus and abdominal wall sometimes forms a long stretched adhesion, which might form a barrier over which a loop of intestine might strangulate. Kelly differentiates between a fixation and a ventro suspension, and claims excellent results in this operation. I may say the difference is not entirely clear to me, unless in the mode of attaching the uterus to the abdominal wall. Some surgeons claim excellent results from ventro suspension, one recording a case of triplets occurring after the operation.

Shortening the round ligaments is another method of operating to relieve prolapse, and is done also in cases of retroversion and for both conditions. It may be done by various methods:

1. In the inguinal canal by the Alexander operation, that can be done with very little risk.

2. By pleating the round ligaments on themselves.

3. By bringing them through the broad ligaments and attaching them to the back of the uterus.

4. Intra-peritoneal shortening of the round ligaments by bringing them up through the peritoneum and bringing them together over the incision and fastening securely.

5. Retro-peritoneal shortening. This operation is, in my opinion, a very good one, as it shortens the round ligaments and attaches them to the anterior abdominal wall and leaves but a single opening into the pelvic cavity, and should obviate the possibility of strangulation of the bowel.

The retro-peritoneal method has been carried out by various authors with very little difference in procedure. There are so many operations to relieve the prolapsus and so many authors who attach their names to the different operations that time does not allow me to discuss all of them pro and con, and it is not my object to criticise too closely the procedures of these great gynecologists. We must suit our operation to the pathological condition we find, and use the method that is nearest to being anatomically correct. By transplantation of the round ligament into the abdominal wall, in one of many different ways, I consider, will give excellent results. Practically speaking it is good, because we make use of the thick end of the round ligament to do work. This type of operation has stood a good clinical test. Any procedure that consists of the doubling up of the thick uterine and of the round ligament in the shortening process and then depends on the terminal frayed out peripheral end to do any holding is anatomically irrational; not only because the round ligament is so diminutive there, but also because it has no attachment or anchorage outside of the parietal peritoneum, except areolar tissue in its course to the inguinal canal. It will yield to any pull that is sufficient to dislodge the parietal peritoneum.

I would like, in closing, to mention a feature which I believe a good many of us overlook in prolapse operations, and that is shortening the utero-sacral ligaments. It may be done by either abdominal or vaginal section. It is indicated wherever these ligaments are overstretched. The typical indication for shortening the utero-sacral ligaments is where the overstretching is extreme and when there is associated with it a posterior enterocele.

